# Demand for long acting contraceptive methods among married HIV positive women attending care at public health facilities at Bahir Dar City, Northwest Ethiopia

**DOI:** 10.1186/s12978-015-0073-0

**Published:** 2015-08-27

**Authors:** Abebaw Addis Gelagay, Digsu Negese Koye, Hedija Yenus Yeshita

**Affiliations:** Department of Reproductive Health, College of Medicine and Health Sciences, University of Gondar, Gondar, Ethiopia; Department of Epidemiology and Biostatistics, College of Medicine and Health Sciences, University of Gondar, Gondar, Ethiopia

## Abstract

**Background:**

The use of long acting contraceptive methods (LACMs) is one of the strategies for preventing mother-to-child transmission (MTCT) of HIV. Studies noted that significant proportion of unintended pregnancy among HIV positive women was due to contraceptive failure mainly of short term contraceptives. This highlights the need to use most effective types of modern contraception, long acting contraceptive. However, studies conducted on demand for long acting contraceptive methods in this particular group of people are scarce in Ethiopia. This study aimed to assess demand for long acting contraceptive methods and associated factors among married reproductive age women attending care at Antiretroviral treatment (ART) clinics in public health institutions at Bahir Dar City, Northwest Ethiopia.

**Methods:**

Institution-based cross-sectional study was conducted among 654 systematically selected women attending care in ART clinics in public health facilities at Bahir Dar city from March to April, 2014. A structured and pretested interviewer administered questionnaire was used to collect data. Data were entered using EPI info version 3.5.3 and then exported to SPSS version 16 for analysis. Descriptive statistics were used to describe the socio-demographic and economic characteristics of the study participants. Logistic regression analyses were employed to identify factors associated with demand for long acting contraceptive methods. Odds ratios with 95 % CI were used to assess the presence and strength of association.

**Results:**

A total of 654 respondents have participated in the study (response rate 99. 09 %). The demand for long acting contraceptive methods was 36.7 % (95 % CI: 33.2 %, 40.6 %).

The odds of demand for LACMs among HIV positive women who were living in urban were three times [AOR = 3.05, 95 % CI: 1.34, 6.89] higher than those who were living in rural. The odds of demand for LACMs among the respondents who were in elementary educational level were two times [AOR = 2.31, 95 % CI: 1.34, 3.99] more likely as compared to those who had no formal education. HIV positive women who had four or more alive children were almost four times [AOR = 3.86, 95 % CI: 1.62, 9.20] more likely to have demand for LACMs than those who had one child or had no child at all. Those who had desire to give birth after 2 years were nearly six times more likely [AOR = 5.68, 95 % CI: 3.05, 11.58] to have demand for LACMs and women who had no birth intension were eight times more likely [AOR = 7.78, 95 % CI: 4.15, 14.58] to have demand for LACMs as compared to those who had intention to have birth within 2 years. Women who had past experience on LACMs had six times more likely [AOR = 6.35, 95 % CI: 4.09, 9.87] to have demand for LACMs than those who hadn’t any experience. The odds of demand for long acting contraceptive methods among HIV positive women who had heard myths about LACMs was 55 % less [AOR = 0.45, 95 % CI: 0.29, 0.68] than those women who hadn’t heard myths.

**Conclusions:**

Demand for long acting contraceptive methods in this study was low. There was high unmet need for LACMs. Myths about LACMs were common in the community and were the major barriers for the promotion and utilization of the methods. Demand creation on LACMs and bringing attitudinal change related to myths through provision of information, education and communication are recommended. Moreover, giving greater attention for rural residents is important.

## Background

Women account for half of the estimated adults living with HIV and AIDS worldwide [1], but higher (59 %) in sub-Saharan Africa [2, 3]. According to Ethiopian Demographic and Health Survey 2011, HIV prevalence among women age 15–49 is 1.9 % and HIV prevalence among pregnant women is 2.4 % [4].

About 90 % of new HIV infection among children under the age of 15 year is as a result of mother-to-child transmission of HIV. World Health Organization (WHO) and the United Nations Joint Programme on HIV/AIDS (UNAIDS) have outlined a 4-element strategy to guide the prevention of mother-to-child transmission (PMTCT) of HIV. The second element of this strategy is prevention of unintended pregnancies among HIV-infected women [5]. Studies have shown that this strategy is at least equally as cost-effective as applying the traditional model of PMTCT services (prevention of mother to child transmission of HIV among HIV positive pregnant women) [6] but it is often overlooked strategy [7–9].

Studies at different parts of the world noted that unintended pregnancy is very high among women living with HIV, for instances, 56 % in Ontario, Canada [10]; 69.2 % in Swaziland [11]; 62 % in South Africa [12, 13]; 62.7 % in Kigali, Rwanda [14]; and 59 % in Kisumu, Kenya [15]. Unintended pregnancy is responsible for 27 % of maternal deaths [16].

The unmet need for family planning among women living with HIV continues to undermine efforts to eliminate new HIV infections among children. Reducing unmet need for family planning will reduce new HIV infections among children and improve maternal health, however it remains high [5]. Un intended pregnancies among women living with HIV takes significant risks for mothers and children. A study in Uganda estimated that even with scale up of antiretroviral based PMTCT, unwanted pregnancy among women with HIV might account for almost a quarter of all HIV positive infants [17]. About one third of unintended pregnancies occur among women accessing contraception mainly who are using short-term methods that require user adherence on a daily or quarterly basis [18]. Poor patterns of short-term hormonal contraceptive use contributed significantly to unintended pregnancy in sub-Saharan Africa [19]. A study conducted in Zimbabwe noted that, among HIV positive women who had unintended pregnancy, about 47.8 % was due to contraceptive failure [20]. Use of LACMs is essential for preventing unintended pregnancies [8]. The use of LACMs is an important strategy for preventing mother-to-child transmission of HIV [9]. Therefore, LACMs, in the context of PMTCT, are the best choices for women living with HIV/AIDS.

A study done in Jinka town, Southern Ethiopia, documented that bout two third (68 %) of HIV positive women had intention to use long acting contraceptive methods though only 7.3 % had used (met need) the methods [21]. A survey in Uganda noted that there was high (90 %) unmet need for a highly effective family planning method in HIV-positive participants [22]. Long-acting contraceptive methods remain out of reach for large numbers of women and couples who want to space or limit child bearing [23]. Empirical evidence indicates that, in some settings, the childbearing intention of HIV positive women has been reduced [22, 24–26]. This implies that women who live with HIV have high contraceptive demand.

Long-acting contraceptive methods are by far the most effective types of modern contraception. LACMs have greater than 99 % protection over a year of use with very low pregnancy rates both in typical users and perfect-users [27, 28]. They are very safe, convenient, and cost-effective in the long-run than short-acting methods since they are less dependent on user adherence and consistent supply chains [27, 28].

While LACMs offer so many comparative advantages, they are not widely used in the general community and are not adequately studied in HIV positive individuals. So, the aim of this study was to assess the demand for LACMs and its associated factors among currently married reproductive age women attending pre-ART or ART services in public health facilities at Bahir Dar City, Northwest Ethiopia. The findings of this study came up with recommendations for the concerned organizations and stake holders which in turn will improve service provision and its uptake/demand. Eventually individuals, families, communities and the nation as a whole will be benefited. Additionally the study findings may be used as an input for other researchers.

## Methods

Institution-based cross-sectional study was conducted among married reproductive age women (15–49 years old women) attending antiretroviral (ART) and pre-antiretroviral (pre-ART) services at Feleg-Hiwot Referral Hospital, and three health centers in Bahir Dar city namely Bahir Dar, Han and Abay health centers.

### Study area and period

The study was conducted at Bahir Dar city administration, Northwest Ethiopia from March to April, 2014. Bahir Dar city is the capital city of Amhara region. It is located at 565 km far from Addis Ababa, the capital city of Ethiopia, to Northwest. According to Amhara regional bureau of finance and economic development (BOFED) 2014 report, Bahir Dar city is divided into 9 sub-cities. There was a total of 288,200 population in the city. From these, 147,597 were females.

In the city, there were one governmental hospital, one private general hospital, ten health centers, more than ten higher and 20 medium private clinics and more than 60 private drug retails. Among the health centers, only five were providing Pre ART and ART services. However, the two health centers (Shembet health center and Tis Abay health center) had begun ART service provision late that they had few (Less than 20 clients) service users. Thus, they were not included in this study. So, the study was conducted at Feleg-Hiwot Referral Hospital, Bahir Dar health center, Han health center and Abay health center.

### Study population

All married reproductive age women who were attending care at ART clinic in public health institutions at Bahir Dar city during data collection period. Reproductive age women who were seriously ill (those who were unable to give the required information) were excluded.

### Sample size determination and sampling procedure

The sample size was computed by using single population proportion formula based on the following assumptions: Since there is no previous study on demand for LACMs and its associated factors on HIV positive women, 50 % was considered as proportion (p) for demand. By considering standard normal distribution the *Z*-value at 95 % confidence level (CI) is 1.96. 4 % were considered as margin of error.

The final sample size planned was 660 after considering 10 % allowance for non response.

The total number of reproductive age (15–49 years) married women who were served in each service delivery points during 1 month period had been estimated based on 1 week preliminary survey. Based on this survey, the estimated number of eligible participants at Felege-Hiwot Referral Hospital, Bahir Dar Health Center, Han Health Center, and Abay Health Center were 3524, 772, 544, and 252 respectively. The sample size was proportionally allocated for each service delivery points and the study participants were selected by systematic random sampling in every seventh interval.

### Operational definitions

#### Long acting contraceptive methods

Modern contraceptive methods that prevent unintended pregnancy for more than 1 year which include Long Acting Reversible contraceptive Methods (LARMs) such as Intra Uterine Devices (IUDs) and sub dermal Implants and permanent contraceptive methods (sterilizations: Tubal ligation and Vasectomy).

#### Demand for long acting contraceptive methods

The sum of long acting contraceptive methods being used (met need), unintended pregnancy (unmet need) and method that is desired but not used due to any reason (unmet need).

#### Unmet need for long acting contraceptive methods

The sum of unintended pregnancies and women having a desire to use long acting method but not used due to any reason.

#### General knowledge on long acting contraceptive methods

If a woman mention at least one of the long acting contraceptive methods and one source.

Myths heard-when women had ever heard any rumor or misperceptions about LACMs.

### Data collection

The data collection tool (questionnaire) was prepared first in English and then translated to a local language (Amharic) and then re-translated back to English language by language experts. Five nurses (who were working in that particular health institutions) for data collection and one public health officer as supervisor were recruited and were given training on the tool and the procedure by principal investigator. The tool was tested on 30 HIV positive women who had getting ART services at Woreta Health center and we made few correction on the tool after the pre-test. In every 7^th^ interval, clients were requested to give the required information and the data collectors had read the verbal consent. Clients, who were volunteered, provided the required information. Data on socio-demographic and economic information, child bearing intention, general knowledge about LACMs, information on the availability of PMTCT services, clinical and contraceptive method related factors were collected using structured and pre-tested interviewer administered questionnaire. The supervisor and the principal investigator supervised the data collection process and looked at data completeness on daily bases.

### Data processing and analysis

The data were checked, coded and entered in to EPI Info version 3.5.3 and exported to SPSS version 16 for analysis. Each variable was first analyzed by using bivariate logistic regression (bivariate analysis) and covariates having *p*-value less than 0.2 was further entered in to multivariable logistic regression model for final analysis. Multivariable analysis was done using backward stepwise logistic regression. In the multivariable analysis, *P*-value less than 0.05 were used as a statistical significant and odds ratio with 95 % CI was used to assess the presence and strength of association between covariates and dependent variable. Hosmer lomeshow test was done to assess fitness of the model. Hosmer lomeshow test greater or equal to 0.05 was considered to ensure goodness of fit of the model.

Ethical clearance was first obtained from the Institutional Review Board (IRB) of Institute of public health, College of Medicine and Health Sciences, University of Gondar. Official permission letter was collected from Amhara Regional Health Bureau and Bahir Dar City Administration Heath Department, and permission letter was sent to the Hospital and Health Centers. Verbal consent was obtained from each study participant. All the study participants were informed about the objective and importance of the study and were also informed about their right not to participate or withdraw from the study at any time.

## Results

### Socio-demographic and economic characteristics of the study participants

A total of 654 women participated in this study with a response rate of 99.09 %. The mean age of the study participants was 31.67 years (SD = 5.46). Majority (85.5 %) of the study participants were Orthodox Christian religion followers. Among the study participants 89.9 % were urban dwellers. The median monthly family income was 52.63 US dollar (Inter Quartile Range = 100 US dollar (Table 1).

**Table 1:** Socio-demographic and economic characteristics of HIV positive women attending care at public health facilities at Bahir Dar City, Northwest Ethiopia, 2014

Variables	Category	Frequency	Percentage
Age (in years)	15–24	49	7.5
	25–34	388	59.3
	35 and above	217	33.2
Religion	Orthodox	559	85.5
	Muslim	65	9.9
	Protestant	27	4.1
	Catholic	3	0.5
Current place of residence	Urban	588	89.9
	Rural	66	10.1
Occupation	House wife	247	37.7
	Daily laborer	130	19.9
	Government employee	127	19.4
	Merchant	124	19.0
	Others^a^	26	4.0
Educational status of women	No formal education	292	44.7
	Primary school (1–8)	136	20.8
	Secondary school (9–10)	110	16.8
	Preparatory, College and above	116	17.7
Educational status of the husband	No formal education	181	27.7
	Primary school (1–8)	155	23.7
	High school (9–10)	140	21.4
	Preparatory, College and above	178	27.2
Family monthly income (US dollar)^b^	<31.63	175	26.8
	31.63–52.64	157	24.0
	52.65–131.58	183	28.0
	>131.58	139	21.2
Ownership of TV & Radio	Yes	537	82.1
	No	117	17.9
Number of children ever born	0–2	480	73.4
	3 and above	174	26.6
Number of alive children	0–1	311	47.6
	2–3	297	45.4
	4 and above	46	7.0

^a^Private employee, farmer, and students

^b^Income was classified according to the quartile classification

### Clinical and method related characteristics of study participants

From all study participants, 533 (81.5 %) were ART users. About 212 (32.4 %) respondents had past experience for LACMs. One fourth, 168 (25.7 %) of the respondents had discussed frequently while slightly more than half, 357 (54.6 %) of the respondents had discussed sometimes about family planning with their partner.

**Fig. 1:**
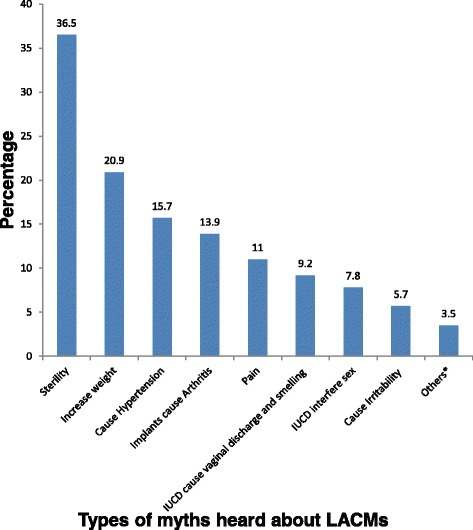
Percentage of respondents who ever heard myths about LACMs by myths type among women attending care at ART clinic in Bahir Dar City, Northwest Ethiopia, 2014. Others*: IUCD can causes heavy menstruation, and causes uterine cancer

Most, 550 (84.1 %), of the respondents had ever heard advertisement about family planning in mass media. Nearly all, 642 (98.2 %), of respondents were aware of at least one LACM and one source (service delivery point for LACMs). From LACMs, Implants (98 %) and IUCD (87 %) were relatively more known methods. About 118 (18.4 %) and 50 (7.8 %) respondents know female sterilization (Tubaligation) and male sterilization (vasectomy) respectively. The sources of information were Mass media, 550 (85.7 %), health professionals, 548 (85.4 %), neighbors, 147 (22.9 %), friends, 94 (14.6 %), husband 76 (11.8 %) and others, 13 (2 %) which include formal education and training. More than half, 364 (55.7 %), of the study participants had ever heard myths about LACMs. Most commonly heard myths include it causes sterility, weight increment and hypertension (Fig. 1).

### Demand for LACMs among study participants

**Fig. 2:**
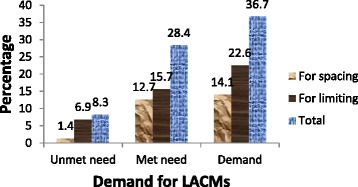
Demand for LACMs among of HIV positive women attending care at public health facilities at Bahir Dar City, Northwest Ethiopia, 2014

The total contraceptive utilization among the study participants was 497 (76 %). From all respondents, 186 (28.4 %) were using long acting methods. The most commonly used long acting contraceptive method was Implant which accounts 153 (23.4 %). The total demand for long acting contraceptive methods was 36.7 % (95 % CI: 33.2–40.6 %). Of which, 186 (28.4 %) was met need and 54 (8.3 %) was unmet need for long acting contraceptive methods. To explain more, among women who had demand for LACMs, the proportion shared by met need (satisfied demand) was 77.5 %. The share of unintended pregnancy for the unmet need for LACMs was 2.4 %. Demand for LACMs for limiting was 22.6 % (Fig. 2).

### Factors associated with demand for LACMs

Urban dweller women were three times more likely [AOR = 3.05, 95 % CI: 1.34, 6.89] to have demand for LACMs as compared to the rural ones. Respondents who were in elementary educational level were two times more likely [AOR = 2.31, 95 % CI: 1.34, 3.99] to have demand for LACMs as compared to those who did not have formal education. Women who had four or more children were four times more likely to have the demand for LACMs as compared to those who had one child or had no child at all [AOR = 3.86, 95 % CI: 1.62, 9.20]. Desire to give birth was found to be a strong predictor of demand for LACMs. Accordingly, women who had desire to give birth after two years were six times more likely [AOR = 5.68, 95 % CI: 3.05, 11.58] to have demand for LACMs and women who had no birth intension were eight times more likely [AOR = 7.78, 95 % CI: 4.15, 14.58] to have demand for LACMs as compared to those who intended to have birth within two years. Women who had past experience on LACMs had six times more likely [AOR = 6.35, 95 % CI: 4.09, 9.87] to have demand for LACMs than those who did not have any experience (Table 2).

**Table 2:** Bivariate and multivariable analysis of variables associated with demand for LACMs in HIV positive women attending care at public health facilities at Bahir Dar City, Northwest Ethiopia, 2014

Independent variables		Demand for LACMs		COR (95 % CI)	AOR (95 % CI)
		Yes	No		
Age	15–24	13	36	1.00	
	25–34	125	263	1.32 (.67–2.57)	
	35 and above	102	115	2.46 (1.23–4.89)	
Residence	Urban	225	363	2.11 (1.16–3.84)	3.05 (1.35–6.89)*
	Rural	15	51	1.00	1.00
Educational Status of Women	No formal education	101	191	1.00	1.00
	Elementary school	56	80	1.32 (.87–2.01)	2.31 (1.34–3.99)*
	High school	39	71	1.04 (.66–1.64)	1.56 (.86–2.80)
	Preparatory, college and above	44	72	1.16 (.74–1.81)	1.63 (.91–2.91)
ART status	Pre-ART	53	68	1.44 (.97–2.15)	
	ART	187	346	1.00	
Number of alive children	0–1	88	223	1.00	1.00
	2–3	123	174	1.79 (1.28–2.51)	1.35 (.86–2.13)
	4 and above	29	17	4.32 (2.26–8.26)	3.86 (1.62–9.20)*
Birth intention	Want within two year	20	148	1.00	1.00
	Want after two year	66	80	6.11 (3.46–10.79)	5.68 (3.05–10.58)*
	No more children wanted	138	137	7.45 (4.42–12.58)	7.78 (4.15–14.58)*
LACMs past experience	Yes	124	88	3.96 (2.80–5.59)	6.35 (4.09–9.87)*
	No	116	326	1.00	
Myths heard	Yes	120	244	.69 (.51–.96)	.45 (.29–.68)*
	No	120	170	1.00	1.00

1.00 = Reference, Hosmer Lomeshow test = 0.757

*p–value <0.05

Women who had heard myths about LACMs was 55 % less likely [AOR = 0.45, 95 % CI: 0.29, 0.68] to have demand for LACMs as compared to those who hadn’t heard myths (Table 2).

## Discussion

Demand for LACMs in the study was 36.7 %. In multivariable analysis, place of residence, educational status of women, the number of alive children they had, birth/reproductive intension, past experience for LACMs, and myths heard about LACMs had statistical significance association with the demand for LACMs.

This study revealed that one third (36.7 %) of HIV positive married reproductive age women had demand for LACMs. This finding was higher than the studies conducted in Southeast Ethiopia (18.1 %) [29], and Central Ethiopia (25.4 %) [30]. The possible reason for the observed difference might be due to variation in fertility intension between HIV positive women and women in the general community-irrespective of their HIV sero-status. Different studies noted that HIV positive women would have less intension to have children [24, 25]. Other possible explanation for this difference might be difference in frequency of contact with health service providers. HIV positive women had frequent visits to health institutions and had more regular contact with health care professionals and hence got opportunities to discuss and received consultations on contraception methods. It was observed that about 98 % of respondents heard about at least one LACMs and one source.

Demand for LACMs in this study was much lower than the study done in Mahabad, Iran (71.35 %) [31]. The possible explanation for this difference might be due to socio-demographic and geographical variation. Another potential reason for this difference might be due to difference in family planning counseling exposure. Since the analysis of the study done in Mahabad, Iran focused on women who were using contraceptive methods at the time of survey, each study participant was exposed for family planning counseling.

The result of this study revealed that the satisfied demand for LACMs was 28.4 %. This was higher than studies done in Gimbie town, West Ethiopia (15.6 %) [32], in Mekelle town (12.3 %) [33], Goba town (8.7 %) [29] and Batu town (3 %) [30]. Geographical variation might be the possible reason for this difference. This might also be related to difference in fertility intention between HIV positive individuals and general community. This finding was found to be lower than the study done in South Africa and Zimbabwe (34 %) [34]. This difference might be due to socio-demographic and economic variation. However, this finding was almost in line with the study done in Mahabad, Iran (27.7 %) [31].

Although the unsatisfied demand for LACMs, 54 (8.3 %), was found lower than the study finding in Batu town (22.4 %) [30] and was almost in line with a study in Goba town (9.4 %) [29] among women in the general population, it is critically important to give due attention to address this gap in HIV positive individuals. Because unintended pregnancy in HIV patients has additional risks such as vertical transmission of the virus in addition to obstetric risks [5] and more serious complications if she ends with induced abortion [16].

In the present study, urban residents were about three times more likely to have demand for LACMs as compared to rural residents. This might be happened because of the difference in general knowledge on LACMs and misperceptions towards LACMs. Another possible reason can be difference in the number of desired children. As it was seen in the 2011 Ethiopian demographic and health survey, urban resident women have relatively less fertility intension than women from rural one [23].

Demand for LACMs was associated with educational status. Women who were in elementary educational level were two times more likely to have demand for LACMs as compared to those who had no schooling. This might be due to difference in detail understanding about the comparative advantages of LACMs. However, association was not found in higher educations.

In this study, there was association between demand for LACMs and the number of alive children they had. The odds of demand for LACMs was four times higher in respondents who had four or more children as compared to those who had one or no child. This association was consistent with studies done in Batu town [30] and Mekelle town [33]. This could be justified that when women have higher number of children, they are more likely to achieve their desire for fertility.

In this study, it was noted that the fertility intention was found to be an important predictor of demand for LACMs. Demand for LACMs was six times more likely in respondents who want birth after two years, and eight times more likely in respondents who want no more children as compared to those who want birth within two years period. This finding is supported by studies conducted at Central Ethiopia [30] and Iran [31].

Past experience for LACMs was strongly associated with demand for LACMs. Those women who had past experience for LACMs were six times more likely to have demand for LACMs than respondents who hadn’t experience. This association was also documented in the study conducted in Goba town [29]. The reason for higher demand for LACMs in those who had past experience might be due to fact that past experience for LACMs may make them familiarized to the methods, make them have more information and they might appreciate method convenience and long term advantages of the methods. Eventually the total and satisfied demand can be high.

Myths or misperceptions towards LACMs were common in the study area (55.7 %) and were found negatively associated with demand for LACMs. Women who heard myths about LACMs were 55 % less likely to have demand for LACMs as compared to those who hadn’t heard. A study conducted in Batu town on general population demonstrated this association [30]. The possible reason is that misperception towards LACMs makes women have negative attitude to LACMs. Which in turn make them choose short acting family planning methods even in those who do not want any more children. This results low demand and utilization for LACMs.

The strength of this study might be addressing a potential area of research which is one of governments’ prioritized/thematic areas of intervention. So as to maintain confidentiality, the data collectors were nurses who were working at the ART and pre-ART services delivery points of the same health institutions. Thus, the possible limitations of this study might be risk for social desirability bias. However, we had strongly informed the data collectors to aware respondents on the importance of factual information and to approach them friendly. Since, there was scarcity of studies on demand for LACMs among HIV positive women, we used studies on the general community irrespective of sero-status.

## Conclusions

The demand for long acting contraceptive methods among currently married HIV positive women was low. The unsatisfied demand for long acting contraceptive methods among currently married HIV positive women was high. Being urban resident, elementary educational level, having four or more alive children, having past experience to LACMs, want to give birth after 2 year, and want no more child had positive association with the demand for LACMs. While myths heard about LACMs was negatively associated with the demand for LACMs.

The authors recommend: Health professionals to provide information, education and communication (IEC) on the multifaceted advantages of the methods and myths about them during family planning counseling service and during health education programs.- The authors also recommend researchers to consider qualitative studies addressing myths on LACMs and their less utilization. Moreover, giving greater attention for rural residents is important.

### Implication of the study

Demand for long acting contraceptive methods among the study participants was 36.7 % (Met need = 28.4 % and Unmet need = 8.3 %). This implies that we need to give more attention to address the unmet need for LACMs. Past experience for LACMs is one of the predictors for demand for LACMs. Most (55.7 %) of the study participants had ever heard myths about LACMs. Myths heard about LACMs had significant association with demand for LACMs. Therefore, provision of information, education and communication is mandatory together with quality family planning counseling service.
